# Evaluating patterns and predictors of symptom change during a three-week intensive outpatient treatment for veterans with PTSD

**DOI:** 10.1186/s12888-018-1816-6

**Published:** 2018-07-27

**Authors:** Alyson K. Zalta, Philip Held, Dale L. Smith, Brian J. Klassen, Ashton M. Lofgreen, Patricia S. Normand, Michael B. Brennan, Thad S. Rydberg, Randy A. Boley, Mark H. Pollack, Niranjan S. Karnik

**Affiliations:** 1 Rush University Medical Center, Chicago, IL 60612 USA; 2 University of California, Irvine, Irvine, CA 92697 USA; 3 Olivet Nazarene University, Bourbonnais, IL 60914 USA

**Keywords:** Veteran, Military, Posttraumatic stress disorder, Combat, Military sexual trauma, Intensive treatment, Cognitive processing therapy, Mindfulness

## Abstract

**Background:**

Intensive delivery of evidence-based treatment for posttraumatic stress disorder (PTSD) is becoming increasingly popular for overcoming barriers to treatment for veterans. Understanding how and for whom these intensive treatments work is critical for optimizing their dissemination. The goals of the current study were to evaluate patterns of PTSD and depression symptom change over the course of a 3-week cohort-based intensive outpatient program (IOP) for veterans with PTSD, examine changes in posttraumatic cognitions as a predictor of treatment response, and determine whether patterns of treatment outcome or predictors of treatment outcome differed by sex and cohort type (combat versus military sexual trauma [MST]).

**Method:**

One-hundred ninety-one veterans (19 cohorts: 12 combat-PTSD cohorts, 7 MST-PTSD cohorts) completed a 3-week intensive outpatient program for PTSD comprised of daily group and individual Cognitive Processing Therapy (CPT), mindfulness, yoga, and psychoeducation. Measures of PTSD symptoms, depression symptoms, and posttraumatic cognitions were collected before the intervention, after the intervention, and approximately every other day during the intervention.

**Results:**

Pre-post analyses for completers (*N* = 176; 92.1% of sample) revealed large reductions in PTSD (*d* = 1.12 for past month symptoms and *d* = 1.40 for past week symptoms) and depression symptoms (*d* = 1.04 for past 2 weeks). Combat cohorts saw a greater reduction in PTSD symptoms over time relative to MST cohorts. Reduction in posttraumatic cognitions over time significantly predicted decreases in PTSD and depression symptom scores, which remained robust to adjustment for autocorrelation.

**Conclusion:**

Intensive treatment programs are a promising approach for delivering evidence-based interventions to produce rapid treatment response and high rates of retention. Reductions in posttraumatic cognitions appear to be an important predictor of response to intensive treatment. Further research is needed to explore differences in intensive treatment response for veterans with combat exposure versus MST.

## Background

According to a recent meta-analysis, approximately 23% of veterans returning from Operation Enduring Freedom and Operation Iraqi Freedom develop posttraumatic stress disorder (PTSD) [1]. Although evidence-based psychotherapies for PTSD such as Cognitive Processing Therapy (CPT) [2, 3] and Prolonged Exposure [4] exist, many veterans do not receive these treatments or fail to receive a sufficient dose of treatment [5]. Research shows that nearly 40% of veterans terminate evidence-based PTSD treatment prior to receiving therapeutic benefit [6]. Several barriers may contribute to low utilization of evidence-based PTSD treatment among veterans including avoidance [7] and poor accessibility of treatment [8, 9].

It is clear that there is a need for greater provision of evidence-based PTSD treatment that is able to address these barriers to its effective utilization. An increasingly popular approach is to deliver these therapies intensively (i.e., daily treatment with patients often living at or near the treatment site during the treatment period) to reduce the susceptibility to external distractions and practical barriers to engaging in treatment, and provide less opportunity for avoidance. Intensive treatments also allow for the integration of multiple treatment modalities, including case management and integrative modalities, which may support treatment adherence and provide more comprehensive care as compared to traditional outpatient therapy. For example, research has shown that the addition of case management services can reduce dropout from cognitive behavioral therapy in vulnerable populations [10].

Residential treatment programs for PTSD typically offer daily treatment over the course of 6–12 weeks with evidence-based treatment (e.g., CPT) delivered twice per week [11–15]. In addition to evidence-based treatment, these programs offer other therapeutic interventions including medication management, psychoeducation, and wellness interventions. Evidence suggests that the delivery of CPT in residential treatment is effective in reducing PTSD and depression symptoms in veterans with different types of trauma (e.g., combat, MST) and comorbidities (e.g., traumatic brain injury, substance abuse) [11–15]. However, the length of time required to complete these programs is often a significant practical barrier for veterans due to concerns about being away from family and work for such a significant period of time.

Several recent studies have shown that more intensive outpatient programs that offer daily evidence-based treatment delivered over the course of 3 weeks are also effective for active duty service members and veterans with PTSD [16, 17]. Lande and colleagues [16] evaluated a three-week intensive outpatient program (IOP) for 39 active duty service members with combat-related PTSD that incorporated daily group and individual cognitive behavioral therapy, coping skill education, medication management, art therapy, and biofeedback. Participation in the IOP resulted in significant reductions in PTSD and depressive symptoms with medium effect sizes [16]. Beidel and colleagues [17] evaluated a 3-week IOP treatment for post 9/11 veterans (*N* = 112) that incorporated daily individual exposure therapy and daily group therapy focusing on behavioral activation, social skills, and anger management. The study revealed large reductions in PTSD symptoms, depression symptoms, guilt, and anger from pre- to posttreatment and these gains were maintained at 6-month follow-up. Moreover, in this study, treatment dropout was much lower than what is typically seen in traditional outpatient treatment.

These findings suggest that IOPs are a promising avenue for delivering evidence-based treatment to veterans and service members with PTSD. However, existing studies have only evaluated key outcomes before and after treatment and have not evaluated the patterns of symptom change of symptom change over the course of treatment. Understanding how veterans improve over the course of intensive treatment is important for establishing the proper dose of treatment, a key question for balancing the feasibility and effectiveness of these programs. Specifically, examining treatment change during the intervention will allow us to determine whether patients plateau and whether shorter interventions would be worthwhile. Moreover, evaluating predictors of treatment response to determine who is most likely to benefit and how they benefit is critical for optimizing the dissemination of intensive treatments.

With women having an increasingly larger presence in the military, it is important to evaluate whether intensive PTSD treatment programs work equally well for men and women and for different trauma types (i.e., combat and military sexual trauma [MST]). One large Veteran’s Administration (VA) study combining data across seven different PTSD intensive treatment programs showed that sex and a history of military sexual assault did not predict treatment outcome [18]. However, this study was limited by the fact that they combined data across very different types of treatment programs and overall the treatment effect sizes were small, suggesting that these programs were not as effective as the intensive outpatient programs [16, 17]. Another VA study examining a 7-week residential PTSD treatment showed that women had a greater decrease in clinician-rated and self-reported PTSD symptoms than men over the course of treatment, but having MST as the index trauma did not predict treatment response [12]. To our knowledge, no studies to date have examined sex and MST as a predictor of treatment response to more condensed intensive outpatient treatment programs.

With respect to how individuals benefit from treatment, current evidence suggests that changes in posttraumatic cognitions may be an important mechanism of cognitive-behavioral treatments for PTSD [19], including Cognitive Processing Therapy [20]. Schumm and colleagues [21] reported that changes in posttraumatic cognitions preceded changes in PTSD symptoms for veterans receiving CPT in a 7-week residential treatment program. However, this study relied on only 3 time points of measurement (pre, mid, post) and no studies have evaluated whether changes in posttraumatic cognitions predict treatment response in more intensive treatment models.

The current study sought to address these important gaps in the literature using effectiveness data from an all-day three-week, cohort-based IOP for service members and veterans with PTSD. The IOP included two treatment tracks (combat-based PTSD and MST-based PTSD), both of which included co-ed cohorts. The goals of the current study were to 1) evaluate patterns of PTSD and depression symptom change over the course of the IOP, 2) examine sex and cohort type (combat vs. MST) as predictors of treatment response, 3) examine changes in posttraumatic cognitions as a predictor of treatment response, and 4) examine whether the relationship between changes in posttraumatic cognitions and treatment response differed by sex or cohort type (combat vs. MST).

## Method

### Intervention

Service members and veterans in this sample participated in a three-week, co-ed, cohort-based IOP designed to treat PTSD secondary to military trauma. The program is housed within a non-VA, mental health clinic that provides services to individuals who served in the U.S. military and their family members free-of-charge. The program runs from 8:00 am to 5:00 pm from Monday through Friday over the course of 3 weeks (15 days of treatment delivered over 19 days). Following the clinical intake evaluation, eligible IOP participants were assigned to one of two IOP tracks (combat or MST) based on their identified index trauma; the treatment tracks ran non-concurrently (for a description of patient flow into the program see Held et al.: Feasibility and acceptability of a three-week intensive outpatient treatment program for service members and veterans with PTSD, in submission). The combat track was designed to meet the needs of veterans experiencing PTSD secondary to combat or warzone stressors. The MST track was offered to veterans with PTSD who experienced military sexual trauma and reported a sexual trauma as their index trauma. Interventions offered in both tracks were largely the same, although some minor modifications were made to address issues specific to each population (e.g., topic-specific psychoeducation sessions for MST). Cohort sizes for both tracks of the program ranged from 5 to 14 participants (*M* = 10.05, *SD* = 2.27), and most cohorts were co-ed. Clinicians were mindful in informing patients about the co-ed groups prior to treatment initiation and worked to ensure that at least 2 individuals of the same sex were in each MST cohort.

The primary IOP intervention components included daily trauma-focused treatment comprised of individual and group CPT [2, 3], as well as daily group integrative health treatment comprised of a mindfulness program that was based on Mindfulness-Based Stress Reduction [22] and yoga. Over the course of the 3 weeks, IOP participants received 15 sessions of individual CPT, 13 sessions of group CPT, 13 sessions of group mindfulness, and 12 sessions of yoga. IOP participants were also assigned daily CPT homework and mindfulness practice. These interventions were modified slightly depending on cohort type (combat vs. MST). For example, the MST track emphasized the esteem and interpersonal difficulties often characteristic of relational trauma.

In addition to these primary intervention components, several secondary intervention components were offered during the three-week IOP program. IOP participants attended experiential and didactic sessions on healthy living that focused on nutrition and physical activity. They also participated in art therapy and groups with a chaplain that focused on making meaning from military service. Psychoeducation sessions focused on common challenges in service members with PTSD such as sleep, pain, relationships, and cognitive health. IOP participants had the option to do up to 6 sessions of acupuncture, meet with a psychiatrist or nurse practitioner for medication management, and meet with a VA Liaison for case management services to assist with continuity of care upon discharge. They were also offered referrals for neuropsychological assessment in cases of suspected traumatic brain injury. Case management services were provided to address legal, financial, or other psychosocial needs. IOP participants attended planned weekend social outings in the city both for enjoyment and as opportunities to practice newly acquired skills (e.g., sports events, city tours). Psychoeducation sessions were offered to family members during the third week of the program in-person or via telehealth. Finally, outreach coordinators worked with participants routinely to ensure that veterans were connected to appropriate aftercare resources (e.g., psychotherapy, pharmacotherapy, vocational services, meditation groups, yoga classes).

#### Cognitive processing therapy

Cognitive Processing Therapy (CPT) is an evidence-based, cognitive-behavioral treatment for PTSD secondary to a range of traumatic experiences, including military trauma and sexual assault [23–25]. The group and individual CPT protocols were structured to accommodate the 3-week format of the IOP (see Appendix A). The content of the individual CPT aligned closely with the CPT protocol [2, 3]. CPT groups were used mainly to practice CPT skills, such as stuck point identification and cognitive restructuring using Socratic dialogue. The initial Impact Statement assignment was modified to facilitate the early identification of assimilated “stuck points.” All participants were encouraged to share their Impact Statement in the groups and group-based Socratic dialogue often led to uncovering specific details about the various index traumas, which appeared to foster group cohesion. Individual and group CPT sessions were conducted by licensed psychologists, psychology postdoctoral fellows, licensed clinical social workers, and licensed professional counselors. All clinicians were trained in CPT by a national subject matter expert. Clinicians were required to participate in official CPT consultation calls following the training and were either rostered on the CPTforPTSD.com website when seeing patients as part of the IOP or working toward becoming rostered. In addition, all clinicians who saw patients as part of the IOP received weekly on-site CPT-consultation from a licensed psychologist with extensive CPT training and experience. Communication between individual and group providers was facilitated through weekly CPT consultation in which the veterans’ stuck points were identified and prioritized for treatment, as well as twice-weekly conference calls among all the IOP providers.

#### Mindfulness based resiliency training

Our intervention, Mindfulness Based Resiliency Training (MBRT) was based on Mindfulness-Based Stress Reduction (MBSR) [22]. Mindfulness, non-judgmental attention on present moment experience, was taught as a way to help participants learn cognitive objectivity, decrease reactivity, and increase affect tolerance. Sessions were delivered by trained MBSR teachers. Content of the MBSR curriculum was maintained but the program was adapted to accommodate the 3-week format of the IOP. Specifically, the 13 sessions were delivered in 75–90 min and one of the sessions in week 2 was a mini retreat of practice without didactics. The yoga (mindful movement) content of MBSR was taught as a separate hour to have sufficient time for the MBRT curriculum and to allow family members who accompanied participants in the last week to participate. The order of the MBSR curriculum content was modified to better align with the CPT curriculum and two sessions of mindful self-compassion [26] were added as a way of helping participants who had experienced moral injury. One session also included an introduction to the mindfulness smartphone apps, Mindfulness Coach [27] and Headspace [28]. The daily home practice sessions, consisting of approximately 15 min of formal and informal mindfulness meditations, were shorter than the standard MBSR home practice.

### Participants

Local and non-local service members and veterans were referred to the program through a variety of sources, including mental health providers/programs, program outreach coordinators, non-profit veteran and social service organizations, other veterans, as well as self-referral. Potential participants completed a comprehensive psychosocial and diagnostic assessment and a series of online screening measures. To be eligible for the IOP, veterans had to report a history of military trauma (e.g., combat or exposure to warzone, military sexual trauma) and to have met the diagnostic criteria for PTSD, which was verified by the Clinician Administered PTSD Scale for DSM-5 - past month version (CAPS-5) [29]. Service members and veterans were ineligible for the program and referred for a higher of level of care if they were experiencing clinical issues that would interfere with their ability to engage in the IOP. Exclusion criteria included active suicidality or homicidality, current engagement in non-suicidal self-harm, active mania or psychosis, active eating disorders, and/or active substance use that would interfere with ability to participate or pose risk of physiological withdrawal. Individuals were also excluded if current medical, legal, or other psychosocial issues would interfere with their ability to fully engage in treatment (for rates and reasons for exclusion see Held et al.: Feasibility and acceptability of a three-week intensive outpatient treatment program for service members and veterans with PTSD, in submission).

The sample for the present study consisted of 191 veterans and service members (94% discharged/retired; 6% on active duty, reserves, or National Guard; henceforth collectively referred to as “veterans”) who completed a 3-week IOP between April 2016 and December 2017. This sample represents 19 cohorts including 12 cohorts of the combat track (*n* = 122; 88.5% male) and 7 cohorts of the MST track (*n* = 69; 18.8% male). On average, veterans were 41.4 years old (*SD* = 9.4, range = 25–69). The majority served in the military after the September 11th terrorist attacks (89.0%), had been deployed (81.5%), and were not local (i.e., greater than 60-mile line-of-sight distance from the mental health clinic; *n* = 170, 89.0%). Other demographic and military characteristics are reported in Table 1.

**Table 1:** Demographic and Military Characteristics

Variable	*n* (%)
Male	121 (63.4)
Ethnicity	
Not Hispanic or Latino	154 (80.6)
Hispanic or Latino	36 (18.9)
Refused	1 (0.5)
Race	
White or Caucasian	130 (68.1)
Black or African American	34 (17.8)
Asian	1 (0.5)
American Indian or Alaskan Native	5 (2.6)
Native Hawaiian or Pacific Islander	3 (1.6)
Other	18 (9.4)
Marital Status	
Single	38 (19.9)
Married/domestic partner	90 (47.1)
Divorced/separated	60 (31.4)
Widowed	3 (1.6)
Last or Current Military Pay Grade	
E1-E3	23 (12.0)
E4-E9	156 (81.7)
Officer/Warrant Officer	12 (6.3)
Branch	
Army/Army Reserve/Army National Guard	124 (64.9)
Air Force/Air Force Reserve/Air National Guard	18 (9.4)
Marine	26 (13.6)
Navy	21 (11.0)
Coast Guard	2 (1.1)
Military Service Status	
Discharged / Retired / Medically Retired	180 (94.2)
Active Duty / Reserves / Inactive Ready Reserve / National Guard	11 (5.8)

*Note*. *N* = 191

### Assessment procedures

As part of the IOP, veterans completed baseline, post-treatment, and daily assessments. Prior to enrolling into the IOP, veterans participated in two 60–90 min clinical intake evaluations with a licensed psychologist, psychology postdoctoral fellow, social worker, or licensed professional counselor. During the intake evaluations, veterans completed a semi-structured psychosocial interview, were assessed for PTSD using the CAPS-5, and were asked to complete a battery of self-report assessments. On average, intake self-report questionnaires were completed 8.25 days (*SD* = 5.15) before they started the IOP program. Veterans were asked to complete additional self-report assessments during the IOP and upon completion of the IOP. All self-report assessments were conducted via Qualtrics [30], a secure online survey tool. This study was approved by the Institutional Review Board at Rush University Medical Center. A waiver of consent was obtained because all assessments were collected as part of routine care procedures.

### Measures

#### Demographics

At intake, veterans provided demographic information, such as age, sex, ethnicity, and education level, as well as military characteristics, such as service branch, last or current military pay grade, service era, and discharge status.

#### Posttraumatic stress disorder

The primary outcome measure for the study was the PTSD Checklist for DSM-5 (PCL-5) [31], a 20-item self-report measure of the DSM-5 symptoms of PTSD. When completing the measure, veterans were directed to rate symptoms in relation to their index trauma. As part of their intake and post-treatment assessments, veterans were asked to rate their PTSD symptoms experienced during the past month. On 9 days during the IOP (every other day with additional assessments to capture the beginning and end of treatment), veterans were asked to report PTSD symptoms experienced during the past week. The past week version of the PCL-5 was used for the daily measures and endpoint scores because it was hypothesized to be more sensitive to any changes that would occur during treatment given that the treatment was shorter than a one-month period. The PCL-5 has been validated and shown to have good internal consistency in samples of veterans and treatment-seeking service members [31–33]. Internal reliability for the past month PCL-5 at intake was .88. Internal reliability of the past week PCL-5 on day 2 of the program was .88.

#### Depression

Depression symptoms were assessed as a secondary outcome measure using the Patient Health Questionnaire – 9 (PHQ-9) [34]. The PHQ-9 is a 9-item self-report measure of DSM-IV criteria for a Major Depressive Episode. The measure asks patients to report on symptoms occurring in the past 2 weeks. The PHQ-9 was assessed during at intake, post-treatment, and on 7 days during the IOP. The measure has been validated and shown to be a have good reliability and internal consistency with a variety of samples, including veterans [35, 36]. Internal reliability for the PHQ-9 at intake was .80.

#### Posttraumatic cognitions

Posttraumatic cognitions were assessed with the Posttraumatic Cognitions Inventory (PTCI) [37]. The PTCI is a 33-item self-report scale that measures trauma-related thoughts and beliefs including negative cognitions about the self, self-blame, and negative cognitions about others and the world. Items are scored from 1 (*totally disagree*) to 7 (*totally agree*). A total score was calculated as the sum of all items with higher scores indicating stronger endorsement of posttraumatic cognitions. The PTCI has demonstrated strong reliability and validity [37] including in military populations with PTSD [38]. Internal reliability for the PTCI at intake was .95.

### Analytic approach for trajectory analysis

Mixed-effects regression models were conducted to examine the trajectory of treatment response over the course of the IOP program due to their less restrictive assumptions regarding the variance-covariance structure, their utility in accommodating some missing measurements across timepoints, and their ability to model individual change over time [39]. Likelihood ratio tests were used for significance testing in mixed effects model comparisons. Some sporadic missing data existed for responses across time points used in longitudinal analyses during the program, though 87.31% of participants utilized in this analysis completed measurements for PTSD symptoms, and 91.04% participants completed depression assessments measurements, during the final two program measurements. Additionally, missingness was not associated with outcome measures at any timepoint or any measured variable. All analyses were conducted in Stata 14 (Statacorp) [40] and Supermix 1.1 (Scientific Software International) [41]. Figures were created in Sigmaplot 13 (Systat Software) [42].

Initial examinations of the correlation structure of PTSD symptom severity (PCL-5) and depression (PHQ-9) scores over time suggested that measurements closer in time were indeed more highly correlated, and that correlations within the same time lags were moderately consistent. This suggested that first-order autoregressive or unstructured covariance pattern models were likely appropriate for errors, which were used for PTSD symptoms and depression scores, respectively, based on Akiake Information Criterion (AIC) analysis. Likelihood ratio tests and AIC values indicated that random intercepts models were a significantly better fit than linear models for both PTSD symptoms and depression (*ps* < .001), and random intercepts and trends models were a significantly better fit compared to random intercepts-alone models (*ps* < .001). A random quadratic trend component also significantly improved fit for PTSD symptom score (*p* < .001) and thus was retained for all mixed effects regression models predicting PCL-5 score that did not include time-varying covariates.^1^ To test the hypothesized prognostic factors (sex and cohort type), we examined the main effects of these variables in the model as well as their interactions with time to determine whether treatment response differed over time based on these variables.^2^

We further examined PTCI scores across the treatment program as a lagged time-varying covariate to assess the relationships between changes in cognitions over the course of the program and PTSD and depression symptoms. PTCI measurements taken on days 2, 4, 9, 11, and 16 served as predictors of both PCL-5 and PHQ-9 outcomes on days 3, 5, 10, 12, and 17 while including time, sex, and cohort type in the models. This also included examining the interaction of the PTCI with time, decomposing within-subject and between-subjects PTCI effects, as well as adjusting for autocorrelation by including the most recent PTSD or depression prior outcome measurements as predictors. Of note, intra-class correlations (ICCs) were greater than .60 for both outcomes, suggesting that a high proportion of unexplained variance existed at the subject level.

## Results

### Treatment engagement

On average, participants completed 13.69 days (*SD* = 1.92) of the 15 days of the program. Of the 191 participants, 176 (92.1%) completed the program. There were no differences in treatment completion by sex (χ^2^ (1) = 1.94, *p* = .163) or cohort type (χ^2^ (1) = 0.06, *p* = .815). Of the 15 participants who did not complete the program, four voluntarily withdrew from the program seemingly due to avoidance, one withdrew due to a family emergency, one withdrew due to perceived lack of improvement, seven were removed due to verbal and physical aggression, one was removed for a medical problem, and one was removed due to failure to attend treatment sessions.

### Pre-post treatment comparison

Paired *t*-tests were conducted to examine changes in symptoms from pre-treatment to post-treatment for treatment completers (see Table 2). Analyses were conducted for the entire sample and by cohort type. Results indicated significant and large reductions in PCL-5 scores (past month *d* = 1.12, past week *d* = 1.40), PHQ-9 scores (*d* = 1.04), and PTCI scores (*d* = 0.75) from pre- to post-treatment. For veterans in the MST cohort, effect sizes were medium to large (*d* = 0.62 to 0.88) whereas effect sizes for veterans in the combat cohorts were large to very large (*d* = 0.85 to 1.81). At post-treatment, 53.4% of veterans no longer met criteria for probable PTSD based on a past-week PCL-5 score ≤ 33 [32]. Rates of remission were significantly different based on cohort type with 62.9% of veterans treated in combat cohorts and 35.7% of veterans treated in MST cohorts no longer meeting criteria for probable PTSD at post-treatment (χ^2^ (1) = 10.81, *p* = .001).

**Table 2:** Paired *T*-tests of Pre- and Post-Treatment Scores for Treatment Completers

Variable	Total Sample				Combat Cohorts				MST Cohorts			
	*n*	Pre-tx *M* (*SD*)	Post-tx *M* (*SD*)	*d*	*n*	Pre-tx *M* (*SD*)	Post-tx *M* (*SD*)	*d*	*n*	Pre-tx *M* (*SD*)	Post-tx *M* (*SD*)	*d*
PCL-5 month	176	57.13 (11.34)	39.78 (18.04)	1.12***	112	57.23 (10.69)	36.94 (17.23)	1.40***	64	56.94 (12.49)	44.77 (18.49)	0.74***
PCL-5 week	157	55.89 (11.67)	33.32 (18.48)	1.40***	104	55.39 (11.25)	29.60 (16.35)	1.81***	53	56.85 (12.51)	40.62 (20.30)	0.88***
PHQ-9	176	17.79 (4.88)	12.05 (5.99)	1.04***	112	17.71 (4.59)	11.03 (5.55)	1.31***	64	17.92 (5.39)	13.83 (6.34)	0.69***
PTCI	176	146.77 (36.05)	115.41 (46.47)	0.75***	112	142.65 (33.25)	109.81 (43.13)	0.85***	64	153.97 (39.74)	125.20 (50.66)	0.62***

*Note*. Pre-tx = Pre-treatment; Post-tx = Post-treatment. PCL-5 month = PTSD Checklist for DSM-5 scores evaluated for the past month. PCL-5 week = PTSD Checklist for DSM-5 scores evaluated for the past week. PHQ-9 = Patient Health Questionnaire – 9. PTCI = Posttraumatic Cognitions Inventory

***p < .001

### Trajectory of treatment response

**Fig. 1:**
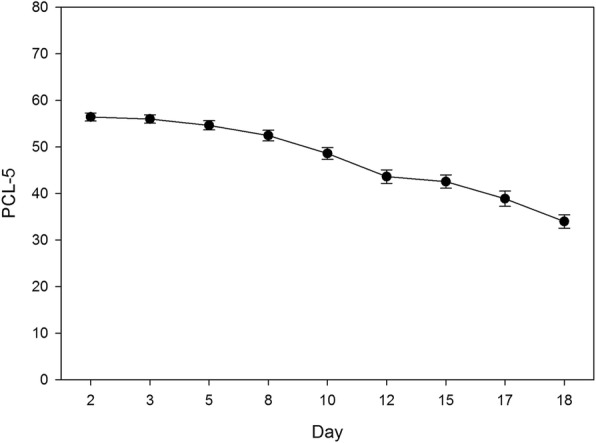
PTSD symptom scores across time during treatment. Note: Error bars represent standard errors. Day represents the day the assessment was taken over the course of the 19 days that participants were in the program (15 treatment days plus 4 weekend days)

**Fig. 2:**
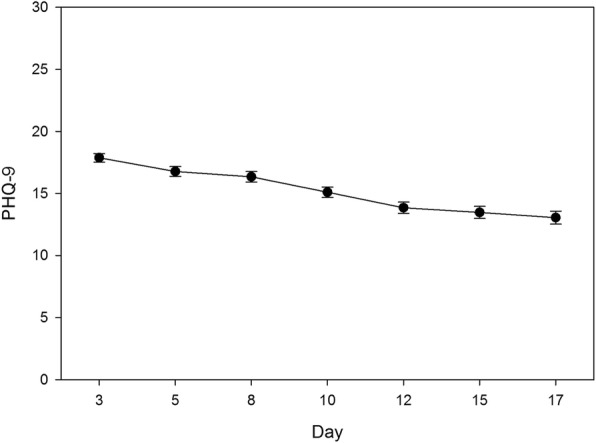
Depression scores across time during treatment. Note: Error bars represent standard errors. Day represents the day the assessment was taken over the course of the 19 days that participants were in the program (15 treatment days plus 4 weekend days)

All participants (completers and non-completers) were included in mixed-effects regression analyses. Examination of time trends in the mixed-effects regression models indicated that significant reductions occurred in both PCL-5 past week and PHQ-9 scores during the course of the treatment program (*ps* < .001; see Table 3). Figures 1 and 2 illustrate the general reduction in PCL-5 and PHQ-9 scores over time, respectively. The significant linear time estimates for PHQ-9 predict a 0.28 point reduction per day in depression score during the treatment program. The quadratic time trend for PCL-5 predicts an accelerating reduction over time for PTSD symptoms from .17 point in the second day to 3.78 point daily reductions at mid-program. These effects remained significant after adjusting for main effects of sex and cohort type. Neither of these covariates were significant predictors of average PCL-5 or PHQ-9 scores.

**Table 3:** Fixed Effects Parameter Estimates for Models of PTSD and Depression Scores

Variable	PCL-5	PHQ-9
	*b (SE)*	*b (SE)*
Time	0.03 (0.30)	−0.28 (0.08)*
Time^2^	− 0.05 (0.01)*	
Sex (male = 0)	0.08 (2.38)	0.83 (1.05)
Cohort Type (MST = 0)	−0.03 (2.39)	−0.32 (1.06)
Sex x Time	0.02 (0.20)	−0.04 (0.08)
Cohort Type x Time	−0.42 (0.20)*	− 0.12 (0.08)

*Note*. *N* = 191. PCL-5 = PTSD Checklist for DSM-5 scores evaluated for the past week. PHQ-9 = Patient Health Questionnaire – 9. Parameter estimates reflect final outcome model estimates, which included all terms. Significance pattern of time trends were the same when covariates were excluded, though parameter estimates differed slightly. The quadratic time component was not significant in models of PHQ-9, and was thus excluded from final models

*p < .05

**Fig. 3:**
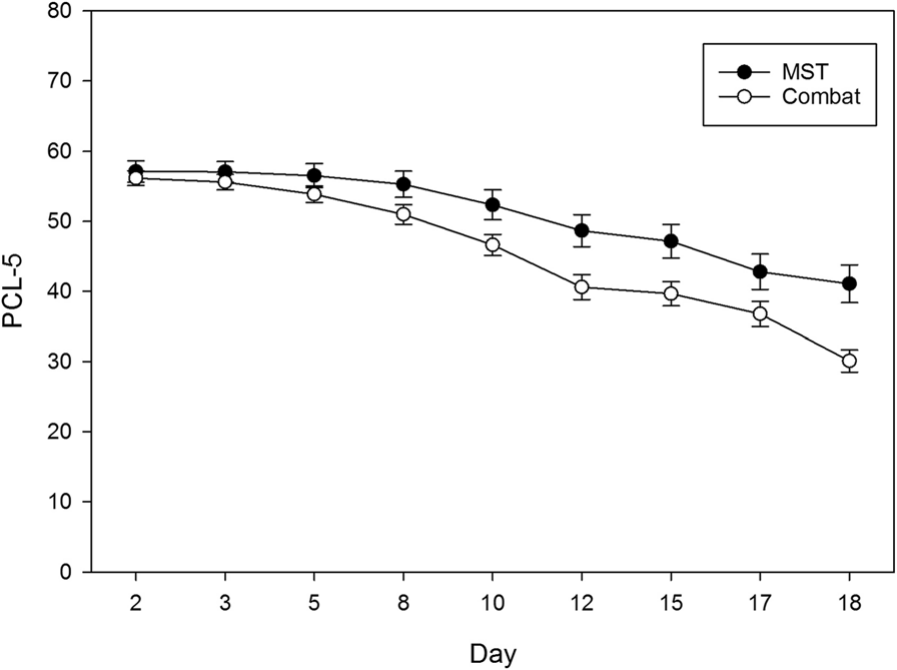
PTSD symptom scores across time by cohort type. Note: Error bars represent standard errors. Day represents the day the assessment was taken over the course of the 19 days that participants were in the program (15 treatment days plus 4 weekend days)

Interactions between time and both sex and cohort type were also examined. A significant cohort type by time interaction was found for PTSD symptoms (*p* = .04) but not for depression (*p* = .38), suggesting differences in PTSD outcome time trends based on cohort type (i.e., combat vs. MST; see Fig. 3). Time by sex interactions were not significant.

### Changes in posttraumatic cognitions as a predictor of changes in PTSD and depression symptoms

Posttraumatic cognitions were examined as a lagged time-varying covariate to assess the relationship between changes in cognitions and changes in PTSD and depression symptoms. PTCI scores were obtained the day prior to PCL-5 and PHQ-9 score assessment, resulting in a one-day lag. In both models, PTCI scores were a significant predictor of subsequent PCL-5 and PHQ-9 scores. Lagged PTCI score remained a significant predictor of both PTSD and depression symptoms following adjustment for autocorrelation using time-lagged PCL-5 or PHQ-9 score (*ps* < .001; see Table 4). Estimates suggest that a 10-point reduction in PTCI score is associated with a PCL-5 decrease of 2.2 and a PHQ-9 decrease of 0.8 (see Table 4).

**Table 4:** Parameter Estimates for PTCI as a Lagged Predictor of PTSD and Depression Scores

Lagged PTCI model variable	PCL-5	PHQ-9
	*b(SE)*	*b(SE)*
PTCI as time-varying covariate	0.22 (0.01)*	0.08 (0.01)*
PTCI disaggregated Within-Subjects	0.32 (0.02)*	0.10 (0.01)*
PTCI disaggregated Between-Subjects	0.21 (0.02)*	0.05 (0.01)*
PTCI adjusting for autocorrelation	0.19 (0.02)*	0.05 (0.01)*
Autocorrelation	0.46 (0.04)*	0.46 (0.04)*

Notes. *N* = 188. Parameter estimates reflect final outcome model estimates, which included time (as previously characterized), sex, and cohort type

*p < .05

Within-subjects and between-subjects effects of PTCI were then disaggregated to examine whether they contributed equally to PTSD and depression outcomes [43]. Separation of these effects resulted in greater model fit for both PCL-5 and PHQ-9 (*p*s < .001), suggesting invariance in the contribution of these effects. Further examination revealed that although both within and between-subjects effects of PTCI were significant predictors of PCL-5 and PHQ-9 scores over time (*ps* < .001), within-subjects reductions in PTCI resulted in greater decreases for both PCL-5 and PHQ-9 relative to cross-sectional between-subjects changes.

Inclusion of PTCI did not alter the significance pattern of sex, cohort type, or the sex by time interaction. However the cohort by time interaction was no longer significant when adjusting for the PTCI. Two-way interactions of sex/cohort and PTCI and three-way interactions of sex/cohort, PTCI, and time were not significant for any outcome. This suggests that the relationship between changes in PTCI and symptom changes were equivalent for men and women and for the different cohort types. The relationships between time-varying PTCI and both PCL-5 and PHQ-9 were also robust to adjustment for autocorrelation (see Table 4).

## Discussion

We evaluated patterns and predictors of symptom change over the course of a 3-week co-ed cohort-based IOP for veterans with PTSD. Consistent with previous research on intensive outpatient programs [17], our intervention resulted in large and clinically meaningful changes in PTSD and depression symptoms. Our effect size for past week PTSD symptoms (*d* = 1.40) was on par with effect sizes established in efficacy trials of psychotherapy for PTSD (*d* = 1.43) [44], suggesting that intensive treatment programs may lead to comparable levels of symptom change as traditional outpatient treatment over a much shorter timeframe (3 weeks compared to 10–12 weeks for typical outpatient treatment). Moreover, adherence and retention in our program was notably high; 92% of patients completed the program and on average, patients completed more than 13 days of the 15-day program. High rates of retention have also been reported for other IOP programs. Beidel and colleagues [17] reported that 89.3% of veterans completed their IOP intervention with only 1.8% of veterans dropping out of treatment and 8.9% of veterans administratively discharged. A recent review describing four IOP programs across the United States, including the current program, reported that across the sites, 95% of veterans completed IOP treatment [45]. The results from these IOP programs compare favorably to previous research showing that nearly 40% of veterans drop out of traditional outpatient PTSD treatment programs [6]. Collectively, these findings suggest that the IOP format can lead to rapid treatment response and help to ensure that patients receive an adequate dose of treatment. However, it is important to note that all of these IOP programs have been administered outside of the VA system, which may also impact treatment adherence and response for a variety of reasons (e.g., differences in patient population, patient expectancy, etc.). Further research is needed to compare IOP and traditional outpatient treatment modalities in a randomized trial to evaluate how treatment delivery format affects adherence and treatment outcome. Moreover, research evaluating the implementation of similar IOP programs within the VA system would help to determine whether this model is equally effective within VA settings.

PTSD symptoms and depression symptoms demonstrated different patterns of change over the course of treatment. Depression symptoms demonstrated a linear decline over the course of treatment whereas PTSD symptoms revealed a quadratic pattern with little symptom change over the first week and an acceleration in symptom reduction over the 2nd and 3rd weeks of the program. Galovski and colleagues [46] evaluated different patterns of symptom reduction over the course of a modified CPT in which the end of treatment was determined by the patient’s individual trajectory. They identified 3 different trajectories of PTSD symptom change, however the trajectory in which patients exhibited high initial symptoms and accelerating change (i.e., patients with the same negative quadratic pattern we observed) was the least common (7.2% of the sample). For depression symptoms, the consistent responders (i.e. patients with the same linear reduction we observed) were one of the more common groups (47.8% of the sample). This may suggest that initial PTSD symptom change may be slower in terms of number of sessions using an intensive approach. This could be due to the time it takes to build trust with providers or the time it takes for patients to consolidate the information they learn and translate that into meaningful changes. Anecdotal evidence suggests that over the first weekend, patients were able to digest the intensive work that was conducted over the first week. Thus, having brief rest periods may be beneficial for the consolidation of gains over the course of intensive treatment. By contrast, depression symptoms may be more liable to change early in intensive treatment given the level of behavioral activation as part of a full-day program. It is also possible that the initiation of an intensive program increases patients’ sense of hope in recovery. Notably, there was no plateau in either PTSD or depressive symptoms at the end of the treatment program. It is possible that a longer treatment program could lead to further symptom reduction, though potentially at the cost of feasibility for patients.

Consistent with previous research in outpatient and residential samples [20, 21], changes in posttraumatic cognitions predicted subsequent changes in PTSD and depression symptoms in our IOP program. Given that CPT directly targets maladaptive cognitions, these results suggest that CPT is an important active ingredient in our integrative IOP treatment, though we are unable to disentangle the effects of the various treatment components that may have impacted cognitions (e.g., mindfulness practice). Regardless of what is driving changes in cognitions, our findings clearly indicate that reductions in posttraumatic cognitions can occur rapidly using an intensive treatment approach and that these changes are meaningfully associated with treatment outcomes. We have shown in the same sample of participants that pre-treatment posttraumatic cognitions predict post-treatment suicidal ideation even when accounting for pre-treatment suicidal ideation, PTSD symptoms, and depression symptoms [47]. Thus, posttraumatic cognitions may be important indicators of treatment response in terms of both symptoms and overall functioning. Future research is needed to evaluate whether posttraumatic cognitions at post-treatment predict long-term functional outcomes and risk for relapse.

Unexpectedly, the combat cohorts revealed a greater reduction in PTSD symptoms over time relative to MST cohorts reflecting an approximate 10-point difference in PTSD symptoms between combat and MST cohorts at treatment endpoint. These findings are inconsistent with previous research showing that MST status did not predict treatment response across several VA intensive PTSD treatment programs [12, 18]. There are several potential explanations that could help to account for these discrepancies. All of the treatments offered at the VA were delivered over longer time period. One possibility is that individuals with MST may not respond as well to treatment over a shortened timeframe. Individuals with MST may have higher rates of interpersonal trauma in childhood [48, 49], which could contribute to more entrenched posttraumatic cognitions that are more difficult to change for individuals with MST compared to those with combat trauma [50]. Notably, the cohort by time interaction was no longer significant after adding posttraumatic cognitions to the model. This may suggest that differences in posttraumatic cognitions across the cohorts drove differences in treatment response; however, it is also possible that this variable became non-significant due to issues of statistical power. Another possibility is that differences in group dynamics affected the cohorts differently. Anecdotally, clinicians reported a larger number of interpersonal issues that arose over the course of group treatment in MST cohorts relative to combat cohorts, which delayed treatment progress. Specifically, interpersonal conflicts among group members in MST cohorts sometimes interfered with the delivery of the CPT group content. It is possible that a greater focus on distress tolerance and interpersonal effectiveness skills early in the IOP program may be beneficial for individuals with MST. Despite the differences across the MST and combat cohorts, it is important to recognize that individuals in the MST cohorts revealed large and clinically meaningful symptom reductions, suggesting that an intensive treatment approach is promising for producing large and rapid symptom reductions for individuals with MST.

Consistent with what has been demonstrated in other intensive PTSD programs [16, 17] as well as traditional outpatient treatment [44], many patients were still symptomatic and did not reach remission at treatment endpoint. These findings may be indicative of several things. First, it is possible that these results are affected by our measurement approach in conjunction with an intensive delivery format. PTSD symptoms are typically assessed over the past month; at treatment endpoint, this would include the time period before the veteran even started treatment. We attempted to correct for this by assessing past week PTSD symptoms and the effect sizes for past week symptoms were notably higher. However, even a past week assessment would mean that veterans would have to account for symptoms occurring before one-third of the treatment was delivered. It is possible that veterans will continue to experience symptom reduction following the IOP treatment without further intervention as they apply newly acquired skills in their home environment and become more confident in their treatment gains. Our findings may also suggest that for many patients, IOP programs can help to stimulate initial symptom reduction, but further outpatient treatment may be needed to achieve remission. Finally, it is also possible that these findings suggest that there may be ways of optimizing our treatment approach to improve outcomes even further, particularly for veterans with MST as their index trauma. For example, booster sessions using telehealth may be indicated.

Our treatment approach is unique in conducting co-ed treatment cohorts; the vast majority of research on intensive treatment for veterans has been done exclusively on single sex groups [11–14]. Although the cohorts were imbalanced with a higher proportion of women in the MST cohorts and a higher proportion of men in the combat cohorts, our findings indicate that male and female veterans benefitted similarly from the IOP. Given the small sample of men with MST, we did not have sufficient power to evaluate whether interactions between sex and MST status predicted treatment response. However, our findings provide preliminary evidence that co-ed cohorts based on trauma type are tolerable and effective for veterans with PTSD.

Several limitations should be taken into consideration when interpreting our results. Because all measures were conducted as part of routine clinical practice, we relied on the use of self-report measures (PCL-5, PHQ-9) as our primary treatment outcomes, rather than gold-standard clinician administered measures such as the CAPS-5. As is typical in effectiveness research, we also did not have a control group in this study. Therefore, we cannot evaluate the degree to which changes over time were due to non-specific treatment components (e.g., therapeutic alliance) versus specific treatment components (e.g., the use of cognitive restructuring techniques). Although all IOP clinicians were CPT trained and received on-site consultation during the IOP, we did not conduct formal treatment fidelity ratings; therefore, we cannot empirically establish the degree to which the CPT protocol was followed. Moreover, our treatment approach was multifaceted with the integration of trauma-focused treatment, wellness, psychoeducation, and case management. We are unable to determine which of these treatment components are necessary for treatment outcomes.

## Conclusions

This study is the first to evaluate patterns and predictors of symptom change over the course of an intensive outpatient PTSD treatment for veterans. This study suggests that IOPs show great promise in delivering full doses of evidence-based treatment and producing rapid and clinically meaningful symptom reduction for different types of veterans including men and women as well as combat and MST trauma survivors. Moreover, our findings suggest that reductions in posttraumatic cognitions may be a key treatment target in CPT-based intensive programs. Given that large amount of subject-level variance observed, more research is needed to determine which factors impact treatment outcomes in this intensive treatment approach to help improve treatment selection and effectiveness.

## Appendix A

**Table 5:** CPT Session Outlines

Day	Group CPT Session Content	Individual CPT Session Content	Homework
Pre-IOP	N/A	• Call client • Introductions • Program overview • Cognitive Processing Therapy ○ 15 daily sessions ○ Trauma-focused ○ Can initially be challenging but we have found it to help with symptom reductions • Questions? • Motivation for treatment • Barriers • Needed support • Check-in about reactions to call	• Call therapist if questions come up
Week 1 Monday	N/A	• Set agenda, make introductions and explain check-in process • PTSD symptoms ○ Intrusions ○ Avoidance ○ Negative alterations in cognitions and mood ○ Hyperarousal • Trauma recovery and Fight-Flight-Freeze response • Cognitive theory • Role of emotions in trauma recovery • Brief review of most traumatic event • Therapy rationale – stuck points • Anticipating avoidance and increasing compliance • Overview of treatment – structured • Discuss group readiness • Check-in about reactions to session	• Write Impact Statement
Week 1 Tuesday	• Agenda, introductions, and check-ins • Group rules • Provide treatment rationale ○ Cognitive theory ○ Types of emotions ○ Biological basis of PTSD • Not everyone responds to trauma the same • Importance of support among group members • First Impact Statement • Reminder: Impact Statement assignment and problem solve completion • Check-in about reactions to session	• Brief check-in • Complete practice assignment review and set agenda • Patient to read Impact Statement • Discuss meaning of Impact Statement • Describe stuck points more fully • Identify stuck points & generate Stuck Point Log • Examine connections among events, thoughts, and feelings • Introduce ABC Worksheets • Check-in about reactions to session	• Complete 3 ABC Worksheets focused on assimilated stuck points
Week 1 Wednesday	• Agenda and check-ins • Share Impact Statements • Introduce connections between events, thoughts, and feelings • Reminder: ABC Worksheet assignment and problem solve completion • Check-in about reactions to session	• Brief check-in • Complete practice assignment review and set agenda • Review ABC Worksheets, further differentiating between thoughts and feelings • Use Socratic questioning on ABC worksheets related to the index event to help patient identify alternative hypotheses • Continue to add to Stuck Point Log • Check-in about reactions to session	• Complete 3 ABC Worksheets focused on assimilated stuck points • Write first Trauma Account
Week 1 Thursday	• Agenda and check-ins • Continue to share Impact Statements • Discuss ABC Worksheets • Introduce Socratic questioning in group, practice challenging assimilated stuck points ○ Up to two patients share • Reminder: ABC Worksheet assignment, Trauma Account assignment, and problem solve completion • Check-in about reactions to session	• Brief check-in • Complete practice assignment review and set agenda • Patient to read full Trauma Account aloud with affective expression • Identification of stuck points • Use Socratic questioning to challenge assimilated stuck points • Explain difference between responsibility and blame • Continue to add to Stuck Point Log • Check-in about reactions to session	• Complete 3 ABC Worksheets focused on assimilated stuck points • Write second Trauma Account
Week 1 Friday	• Agenda and check-ins • Share thoughts and feelings about writing Trauma Account • Practice challenging assimilated stuck points ○ Up to two patients share • Reminder: ABC Worksheet assignment, Trauma Account assignment, and problem solve completion • Check-in about reactions to session	• Brief check-in • Complete practice assignment review and set agenda • Patient to read full Trauma Account aloud with affective expression; help identify differences between first and second account • Introduce Challenging Questions Worksheet • Use Socratic questioning to challenge assimilated stuck points • Check-in about reactions to session	• Complete 3 Challenging Questions Worksheet
Week 2 Monday	• Agenda and check-ins • Discuss re-writing Trauma Account • Assess for and normalize strong emotions at this phase of therapy • Practice challenging assimilated stuck points ○ Up to two patients share • Reminder: Challenging Questions Worksheet assignment and problem solve completion • Check-in about reactions to session	• Brief check-in • Complete practice assignment review and set agenda • Review Challenging Questions Worksheet ○ Focus on assimilated stuck points • Continue cognitive therapy for stuck points • Introduce Patterns of Problematic Thinking Worksheet • Check-in about reactions to session	• Complete 1 Patterns of Problematic Thinking Worksheet
Week 2 Tuesday	• Agenda and check-ins • Practice challenging assimilated stuck points ○ Up to two patients share • Reminder: Patterns of Problematic Thinking Worksheet assignment and problem solve completion • Check-in about reactions to session	• Brief check-in • Complete practice assignment review and set agenda • Review Patterns of Problematic Thinking Worksheet ○ Complete challenging Questions if patient is still struggling with content • Continue cognitive therapy for stuck points • Introduce Challenging Beliefs Worksheets • Introduce first of five problem areas: Safety • Check-in about reactions to session	• Complete 3 Challenging Beliefs Worksheets • Review Safety Module
Week 2 Wednesday	• Agenda and check-ins • Practice challenging assimilated stuck points ○ Up to two patients share • Reminder: Challenging Beliefs Worksheet assignment and problem solve completion • Check-in about reactions to session	• Brief check-in • Complete practice assignment review and set agenda • Review Challenging Beliefs Worksheet ○ Address remaining assimilated stuck points or Safety stuck points • Help patient confront problematic cognitions and generate alternative beliefs using the Challenging Beliefs Worksheet • Assign Challenging Beliefs Worksheets • Introduce second of five problem areas: Trust • Check-in about reactions to session	• Complete 3 Challenging Beliefs Worksheets • Review Trust Module
Week 2 Thursday	• Agenda and check-ins • Practice challenging assimilated stuck points ○ Up to two patients share • Reminder: Challenging Beliefs Worksheet assignment and problem solve completion • Check-in about reactions to session	• Brief check-in • Complete practice assignment review and set agenda • Review Challenging Beliefs Worksheet ○ Address remaining assimilated stuck points or Trust stuck points • Help patient confront problematic cognitions and generate alternative beliefs using the Challenging Beliefs Worksheet • Assign Challenging Beliefs Worksheets • Introduce third of five problem areas: Power/Control • Check-in about reactions to session	• Complete 3 Challenging Beliefs Worksheets • Review Power / Control Module
Week 2 Friday	• Agenda and check-ins • Practice challenging assimilated/over-accommodated stuck points ○ Up to two patients share • Reminder: Challenging Beliefs Worksheet assignment and problem solve completion • Check-in about reactions to session	• Brief check-in • Complete practice assignment review and set agenda • Review Challenging Beliefs Worksheet ○ Address remaining assimilated stuck points or Power/Control stuck points • Help patient confront problematic cognitions and generate alternative beliefs using the Challenging Beliefs Worksheet • Assign Challenging Beliefs Worksheets • Introduce fourth of five problem areas: Esteem • Check-in about reactions to session	• Complete 3 Challenging Beliefs Worksheets • Review Esteem Module
Week 3 Monday	• Agenda and check-ins • Practice challenging assimilated/over-accommodated stuck points ○ Up to two patients share ○ Focus on trauma themes • Reminder: Challenging Beliefs Worksheet assignment and problem solve completion • Check-in about reactions to session	• Brief check-in • Complete practice assignment review and set agenda • Review Challenging Beliefs Worksheet ○ Address remaining assimilated stuck points or Esteem stuck points • Help patient confront problematic cognitions and generate alternative beliefs using the Challenging Beliefs Worksheet • Assign Challenging Beliefs Worksheets • Introduce fifth of five problem areas: Intimacy • Initiate contact with community provider in patient’s area • Check-in about reactions to session	• Complete 3 Challenging Beliefs Worksheets • Review Intimacy Module
Week 3 Tuesday	• Agenda and check-ins • Practice challenging assimilated/over-accommodated stuck points ○ Up to two patients share ○ Focus on trauma themes • Reminder: Challenging Beliefs Worksheet assignment and problem solve completion • Check-in about reactions to session	• Complete practice assignment review and set agenda • Review Challenging Beliefs Worksheet ○ Address remaining stuck points • Help patient confront problematic cognitions and generate alternative beliefs using the Challenging Beliefs Worksheet • Assign Challenging Beliefs Worksheets • Continue to establish contact with community provider in patient’s area • Check-in about reactions to session	• Complete 3 Challenging Beliefs Worksheets
Week 3 Wednesday	• Agenda and check-ins • Practice challenging assimilated/over-accommodated stuck points ○ Up to two patients share ○ Focus on trauma themes • Reminder: Challenging Beliefs Worksheet assignment and problem solve completion • Check-in about reactions to session	• Complete practice assignment review and set agenda • Review Challenging Beliefs Worksheet ○ Address remaining stuck points • Help patient confront problematic cognitions and generate alternative beliefs using the Challenging Beliefs Worksheet • Assign Final Impact Statement • Continue to establish contact with community provider in patient’s area • Check-in about reactions to session	• Final Impact Statement
Week 3 Thursday	• Agenda and check-ins • Discuss Final Impact Statement • Involve patients in reviewing the course of treatment and patient’s progress • Help identify goals for the future and delineate strategies for meeting them • Check-in about reactions to session/program	• Complete practice assignment review and set agenda • Review Challenging Beliefs Worksheet ○ Address remaining stuck points • Help patient confront problematic cognitions and generate alternative beliefs using the Challenging Beliefs Worksheet • Patient to read Final Impact Statement • Involve patient in reviewing the course of treatment and patient’s progress • Help identify goals for the future and delineate strategies for meeting them • Continue to establish contact with community provider in patient’s area • Check-in about reactions to session	N/A
Week 3 Friday	N/A - Graduation	• Review course of treatment and patient progress • Go over discharge plan • Assess patient goals for the future • Continue to establish contact with community provider in patient’s area • Review referrals and plan for aftercare	N/A - Graduation

## Footnotes

1 Quadratic time effects were non-significant for both PCL and PHQ outcomes when including lagged time-varying PTCI as a covariate, and were thus excluded from model estimates.

2 To examine clustering by cohort, three-level mixed models were also examined. However, because trends and significance patterns for time and covariates of interest were nearly identical, and due to our interest in including cohort type (MST and combat), only two-level models were reported here.
